# Natural Deep Eutectic Solvent‐Assisted Construction of Silk Nanofibrils/Boron Nitride Nanosheets Membranes with Enhanced Heat‐Dissipating Efficiency

**DOI:** 10.1002/advs.202403724

**Published:** 2024-07-25

**Authors:** Yang Wang, Zhaohui Yang, Bingzheng Jia, Lan Chen, Chuanyu Yan, Feng Peng, Tiancheng Mu, Zhimin Xue

**Affiliations:** ^1^ Beijing Key Laboratory of Lignocellulosic Chemistry State Key Laboratory of Efficient Production of Forest Resources Beijing Forestry University Beijing 100083 China; ^2^ School of Chemistry and Life Resources Renmin University of China Beijing 100872 China

**Keywords:** boron nitride nanosheets, deep eutectic solvents, hybrid nanocomposite membranes, silk nanofibrils, thermal conductivity

## Abstract

Natural polymer‐derived nanofibrils have gained significant interest in diverse fields. However, production of bio‐nanofibrils with the hierarchical structures such as fibrillar structures and crystalline features remains a great challenge. Herein, an all‐natural strategy for simple, green, and scalable top‐down exfoliation silk nanofibrils (SNFs) in novel renewable deep eutectic solvent (DES) composed by amino acids and D‐sorbitol is innovatively developed. The DES‐exfoliated SNFs with a controllable fibrillar structures and intact crystalline features, novelty preserving the hierarchical structure of natural silk fibers. Owing to the amphiphilic nature, the DES‐exfoliated SNFs show excellent capacity of assisting the exfoliation of several 2D‐layered materials, i.e., h‐BN, MoS_2_, and WS_2_. More importantly, the SNFs‐assisted dispersion of BNNSs with a concentration of 59.3% can be employed to construct SNFs/BNNSs nanocomposite membranes with excellent mechanical properties (tensile strength of 416.7 MPa, tensile modulus of 3.86 GPa and toughness of 1295.4 KJ·m^−3^) and thermal conductivity (in‐plane thermal conductivity coefficient of 3.84 W·m^−1^·K^−1^), enabling it to possess superior cooling efficiency compared with the commercial silicone pad.

## Graphical abstract

Natural deep eutectic solvent‐exfoliated silk nanofibers with highly intact natural hierarchical structure can effectively assist liquid‐phase exfoliation of 2D materials via hydrogen bonding and hydrophobic–hydrophobic interactions. The formed dispersion of silk nanofiber and boron nitride nanosheet can self‐assembly into nanocomposite membranes with excellent mechanical strength and enhanced heat dissipation properties.

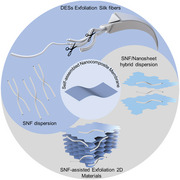

## 1 Introduction

Silkworm silk fibers, an attractive natural biopolymer, possess combined merits of exceptionally strong mechanical properties, scalable renewability, flexible processability, and splendid amphiphilicity.^[^ ^1^ ^]^ Natural silkworm silk fibers are consisted of thick microfibrils (20–200 nm), which are assembled from bundles of interlocking nanofibers with a diameter of ≈3 nm.^[^ ^2^ ^]^ Within each silk nanofiber (SNF), the hydrophobic *β*‐crystallites embedded in hydrophilic amorphous matrix co‐assemble into a unique hierarchical and semicrystalline network structure with inherent amphiphilicity.^[^ ^3^ ^]^ The excellent properties of silk fibers were closely related to the hierarchical and semicrystalline structure, especially the unique spatial arrangement of secondary structure at mesoscale and nanoscale. Generally, small changes in secondary structure at either scale can lead to notable changes in the properties of silk fibers.^[^ ^4^ ^]^ Compared with silkworm silk microfibrils, SNFs with the natural hierarchical structure have greater potential for practical applications. Thereby, the generation of SNFs from silkworm silk microfibrils has attracted significant interest.

Currently, bottom‐up self‐assembly and top‐down physical or chemical nanofibrillation have been developed to fabricate SNFs. The bottom‐up self‐assembly methods for the preparation of SNFs are mainly based on silk fibroin solution, which often involved complex manufacturing requirements and environmental‐unfriendly or expensive solvents.^[^ ^5^ ^]^ More unfortunately, the silk fibroin is severely degraded to molecular level during its dissolution process, resulting in the loss of the natural hierarchical structure in the regenerated SNFs. As a result, the properties of the manufactured materials using the prepared SNFs from the bottom‐up self‐assembly are inferior to those of natural silk fibers. Compared with the regenerated SNFs from silk solutions, the SNFs derived by direct top‐down exfoliation of natural silk fibers using weaker solvent systems reserve the natural microstructure and high crystallinity of natural silk fibers. However, the developed solvent systems for direct exfoliation of silk have several limitations, including toxic reagents, complex/long‐term processes, strict storage conditions, and short stabilization times for SNFs dispersions. These limitations extremely hamper the large‐scale fabrication of SNFs by the current‐developed top‐down exfoliation systems. For instance, although SNFs with excellent water stability and reprocessability could be extracted, highly toxic hexafluoroisopropanol was employed.^[^ ^6^ ^]^ NaOH/urea solvent could produce silk nanoribbons consisting of single *β*‐sheet and amorphous silk molecules, but a long and repeated freezing/thawing process was required.^[^ ^7^ ^]^ Besides, cumbersome steps were required for the production of SNFs in 2,2,6,6‐tetramethy‐pipelidine‐l‐oxyl radical/NaBr/NaClO solvent systems.^[^ ^8^ ^]^ Moreover, the SNFs dispersion prepared by the formic acid/LiBr solvent system must be stored at 4 °C to avoid the gelation,^[^ ^9^ ^]^ and the SNFs generated from formic acid/CaCl_2_ solvent were difficult to keep a stable aqueous dispersion with a stabilization time of less than 6 h.^[^ ^10^ ^]^ Thus, construction of innovative solvent systems to direct exfoliate silk fibers into SNFs with highly intact natural hierarchical structure has become an important research topic. As one class of green and chemically tailorable solvents, deep eutectic solvents (DESs) with special microstructure and physicochemical properties have been designed by properly combining various hydrogen bond acceptors/donors for biopolymers dissolution or fibrillation.^[^ ^11^ ^]^ In the fibrillation of silk fibers, DESs attacked the amorphous regions of natural silk fibers to exfoliate silk fibers into micrometer/nanometer scale segments, and the natural structure of silk fibers was retained maximumly. It was reported that DESs composed by guanidine hydrochloride and urea could effectively exfoliate natural silk fibers to generate SNFs with controllable diameters and multi‐compatible stability in varied polar solvents. Importantly, the formed SNFs preserved the fibrillar structure, allowing the generation of free‐standing SNFs membranes with a nanoporous structure.^[^ ^12^ ^]^ In spite of the attractive progress, the application of DESs in top‐down exfoliation of silkworm silk fibers is still in a nascent stage.^[^ ^13^ ^]^ Further expansion of novel DESs, that can be used for direct exfoliation of silk fibers to produce SNFs with similar secondary structure and crystallinity to the native silk, is a prerequisite for subsequent diverse applications of the generated SNFs.

Monolayer and few‐layered 2D nanosheets can be achieved from the exfoliation of the corresponding bulk layered materials, which exhibit a strong hydrophobic character, thus severely hampering the scalable liquid‐phase exfoliation/dispersion process for producing nanosheet in aqueous phase.^[^ ^14^ ^]^ Moreover, due to the lack of hydrophilic groups on the exfoliated nanosheet, reaggregation was one of the main challenge for the water‐dispersed nanosheet.^[^ ^15^ ^]^ Generally, amphiphilic additives can assist the exfoliation of bulk layered materials and improve the stable dispersion of hydrophobic nanosheets in water.^[^ ^16^ ^]^ As alternatives to traditional amphiphilic additives, various proteins have been used to assist in the exfoliation of 2D‐layered materials and defect‐free 2D nanosheets could be successfully obtained and steadily dispersed in aqueous solutions.^[^ ^17^ ^]^ However, rare sources and expensive prices for these proteins restrict their large‐scale utility for the production. From the discussions above, we deduce that amphiphilic natural biopolymers may be the promising renewable alternatives to conventional surfactants for liquid‐phase production/dispersion of 2D nanosheets.

Herein, the development of green and scalable production of SNFs with natural hierarchical structures as well as the exploration of natural dispersants for 2D materials were of great significance for the enrichment of industrial routes and the sustainable development of the society. We designed robust natural DESs composed by amino acid and sorbitol for the facile, scalable, and direct top‐down exfoliation of natural silk fibers into nanofibrils. Thanks to the excellent swelling ability of DESs, the exfoliated SNFs might preserve its natural hierarchical structures and amphiphilic property, demonstrating excellent ability in 2D material exfoliation and dispersion, and providing strong mechanical properties for SNFs‐based composites. Interestingly, the resulted DES‐exfoliated SNFs retained highly intact secondary structures and the excellent amphiphilicity of natural silk fibers. These features afforded the generated SNFs being a green and effective additive for 2D‐nanomaterials (using boron nitride as an example) exfoliation and dispersion. More attractively, SNFs/BNNSs (boron nitride nanosheets) nanocomposite membranes generated from the SNFs‐assisted dispersion solution of BNNSs exhibited excellent mechanical strength and thermal conductivity, allowing their potential application for heat spreaders in the electronic devices.

## 2 Results and Discussion

**Figure 1:**
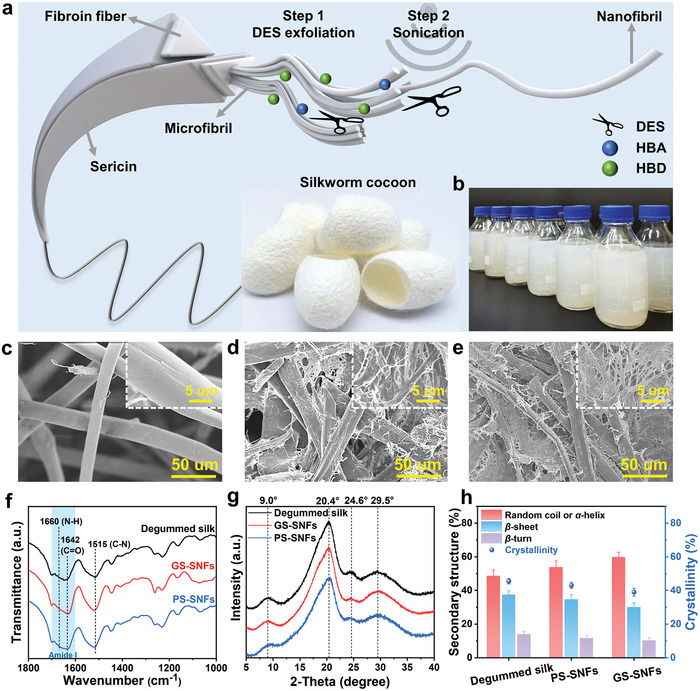
a) Schematic illustration for nanofibrillation of silk fibers by DES‐exfoliation and ultrasonic disintegration. b) Mass production of SNFs. SEM images of the c) degummed silk fibers, d) GS DESs‐exfoliated SNFs, and e) PS DESs‐exfoliated SNFs, inset shown corresponding partial enlargement. f) FTIR spectra, g) XRD patterns, h) the crystallinity and the secondary structures content of degummed silk and DESs‐exfoliated SNFs. Data presented as mean ± SD, n = 3.

The two‐step “top‐down” process of exfoliating natural silk fibers into SNFs were illustrated in **Figure** **1**a, which was mainly consisted of DESs chemical pretreatment and mechanical (sonication) disintegration. Initially, two natural DESs systems with low melting point (<−15 °C) and superhigh thermal stability (>200 °C), i.e., natural L‐glutamic acid/D‐sorbitol (GS) and L‐proline/D‐sorbitol (PS), were successfully prepared (Figure S1, Supporting Information) to exfoliate silk fibers to generate micron/nano‐scale segments or micro‐gaps between the nanofibrils, which were further disintegrated into homogeneous nanofibers by sonication. The microstructure changes during the DESs exfoliation process were examined by scanning electron microscopy (SEM). From the SEM images of the silk fibers, the diameters of the silk fibers decreased significantly and a considerable number of nanoscale fibrils were exposed on the surface of silk fibrils (Figure 1d,e) in comparison with the untreated silk fibrils (Figure 1c), verifying that silk fibrils could be successfully exfoliated into nanofibrils by amino acid/sorbitol‐based DESs. The ability of the determined DESs to exfoliate silk fibers was mainly attributed to the formation of hydrogen bonding between DESs and silk,^[^ ^12^, ^13^ ^]^ which would weaken or partially destroy the strong hydrogen bond interactions and hydrophobic interactions in silk, consequently disrupting the intramolecular interactions and resulting in swelling and loosening of the silk fibers. Homogeneous and well‐dispersed SNFs aqueous suspensions could be prepared by ultrasonication of the DESs‐treated silk fibers (400 W, 4 h), followed by centrifugation to remove the un‐exfoliated aggregates (Figure 1a). Moreover, scaled‐up fabrication of SNFs could be easily achieved by increasing the amount of the DESs/silk fibers (Figure 1b). Typically, 1 g of degummed silk fibers could produce 500 mL SNFs aqueous dispersion after liquid exfoliation by GS‐DESs or PS‐DESs and ultrasonication, and the concentrations of SNFs could reach ≈1.15 and 0.49 mg mL^−1^ using GS‐DESs and PS‐DESs, respectively. Correspondingly, the yields of SNFs were ≈57.5% and 24.5%. Besides, the concentration and yield of DES‐exfoliated SNFs decreased with the increasing centrifugation rates (Table S1, Supporting Information). Additionally, the un‐exfoliated silk fibers could be further pretreated and exfoliated to reprepare SNFs, thus improving the total yield of SNFs from 57.5% to 70.3%. The obtained GS‐exfoliated SNFs at the different centrifugation rates (i.e., 2000, 5000, and 8000 rpm) were further characterized by transmission electron microscope (TEM) and anatomic force microscopy (AFM). Based on a statistical analysis of the TEM and AFM images (Figure S2a,b, Supporting Information), the GS‐DESs‐exfoliated SNFs exhibited a wide range of contour lengths and diameter distribution that decreased with the increasing centrifugation rates, resulting in controllable contour lengths of 0.1–2.6 µm and diameters of 18–100 nm. Specifically, the average lengths of GS‐SNFs‐2000, GS‐SNFs‐5000, and GS‐SNFs‐8000 were 0.99, 0.78, and 0.56 µm (Figure S2c, Supporting Information), respectively, corresponding to an average diameter of 55.1, 41.5, and 31.4 nm (Figure S2d, Supporting Information). DESs‐exfoliated SNFs had hydrophilic function groups with negative surface charge and showed a high value of zeta potential (−31.5 mV) in neutral aqueous dispersion, which impart it to form a homogeneous dispersion exhibiting obvious Tyndall effect (Figure S3, Supporting Information).

To investigate whether chemical modification and de‐crystallization of silk occurred during the DESs exfoliation, Fourier‐transform infrared spectroscopy (FT‐IR) and X‐ray diffraction (XRD) techniques were conducted. As shown in FT‐IR spectra (Figure 1f), the signal patterns of degummed silk, GS‐DESs‐exfoliated and PS‐DESs‐exfoliated SNFs appeared to be rather similar, indicating that the basic molecular conformations of silk were remained after the DESs treatment. For amide I, the major peaks at 1642, 1660, and 1699 cm^−1^ were assigned to *β*‐sheet, random coil/*α*‐helix, and *β*‐turn structures, respectively.^[^ ^18^ ^]^ The absorption peaks at 1642, 1660, and 1515 cm^−1^ were indicative of the C═O bond vibration of amino acid residues in the *β*‐sheet structure, and the N─H and C─N bond vibration of amino acid residues in the *α*‐helix structure, respectively.^[^ ^19^ ^]^ In addition, the heavy chain polypeptide of silk fibroin mainly consists of 12 hydrophobic repetitive domains and 11 hydrophilic nonrepetitive domains, which form highly ordered crystalline regions (*β*‐sheet structure) and amorphous regions (random coil or *α*‐helix structures) in the fibroin, respectively.^[^ ^20^ ^]^ Therefore, the secondary structure content of DESs‐exfoliated SNFs was a valid basis for verifying the preservation of natural amphiphilicity during the DESs exfoliation process. The contents for secondary structures in the DESs‐exfoliated SNFs could be further quantified from the deconvolution of amide I bonds in FT‐IR spectra (Figure S4, Supporting Information), and the corresponding contents of *β*‐sheet, *β*‐turn, and random coil/*α*‐helix were shown in Figure 1h. Compared to natural silk fibers, the contents of *β*‐sheet and *β*‐turn slightly decreased and the content of random coil/α‐helix slightly increased after DESs exfoliation, further confirming that DESs‐exfoliated SNFs had abundant and highly intact secondary structures. GS‐DESs‐exfoliated SNFs contained lower *β*‐sheet (30%) and higher random coil/*α*‐helix (59.8%) compared to the corresponding conformations of PS‐DESs‐exfoliated SNFs, which implied that GS‐DESs had a stronger ability to break hydrogen bonding in silk fibers than PS‐DESs, thus resulting in a higher yield of SNFs by using GS‐DESs as the exfoliation solvent. Besides, similar XRD patterns were observed for degummed silk, GS‐DESs‐exfoliated and PS‐DESs‐exfoliated SNFs (Figure 1g), and four characteristic peaks at ≈9.0°, 20.4°, 24.6°, and 29.5° were detected.^[^ ^21^ ^]^ Deconvolutions of XRD patterns were conducted to calculate the relative crystallinity of SNFs (Figure S5, Supporting Information). The crystallinity of SNFs exfoliated by GS‐DESs and PS‐DESs was 39.5% and 43.2%, respectively, which slightly decreased compared with that of the degummed silk (45.5%). The decreasing trend of the crystallinity was consistent with that of the *β*‐sheet content. Based on the discussions above, we could conclude that the prepared amino acid/sorbitol DESs were efficient solvents for exfoliation of natural silk fibers into nanofibrils with highly intact secondary structures. The amino acid/sorbitol DESs as a mild solvent, which only effectively swelling without dissolving the silk fibers and thus could maintain the hierarchical structures such as fibrillar structures and crystalline features from natural silk in the exfoliation step.^[^ ^12^ ^]^ The demonstrated DESs‐assisted exfoliation for fabricating SNFs with natural hierarchical structures may provide a useful idea for designing solvent systems for top‐down exfoliation of SNFs and pathway for manufacturing stronger SNFs‐based materials for various applications.

**Figure 2:**
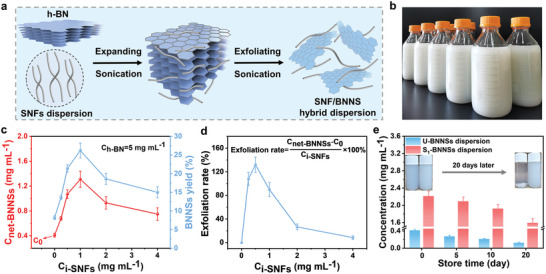
a) Scheme of the SNFs‐assisted liquid exfoliation of h‐BN. b) Large‐scale production of S_1_‐BNNSs dispersion with a net BNNSs concentration of 1.31 mg·mL^−1^. c) Net concentrations (C_net‐BNNSs_) and yields of BNNSs in S_n_‐BNNSs dispersions. d) The exfoliation rates of BNNSs. e) Concentration changes of U‐BNNSs dispersions and S_1_‐BNNNs hybrid dispersions under room temperature storage, the insets were the photographs of the dispersions U‐BNNSs and S_1_‐BNNSs before and after 20 days of standing. Data presented as mean ± SD, n = 3.

In the molecular skeleton of SNFs, there were abundant hydrophilic polar ‐OH, ‐COOH and ‐NH_2_ groups in the amorphous region, which could form hydrogen bonding interactions with the aqueous phase.^[^ ^22^ ^]^ In this work, the water contact angle for the surface of DESs‐exfoliated SNFs membrane was 56.0±3.4° at 0 s, indicating that the DESs‐exfoliated SNFs were moderately hydrophilic (Figure S6, Supporting Information). A large number of hydrophobic *β*‐sheet crystallites were also preserved in the SNFs, which made SNFs attach to the surface of bulk 2D‐nanomaterials through hydrophobic interaction.^[^ ^23^ ^]^ The amphiphilic nature allowed DESs‐exfoliated SNFs to potentially be effective agents for exfoliating and dispersing 2D‐nanomaterials. To test the feasibility of SNFs as exfoliating and dispersing agents, liquid‐phase exfoliation of pristine hexagonal boron nitride (h‐BN) with a concentration of 5 mg mL^−1^ was conducted in the aqueous dispersions of SNFs (**Figure** **2**a). After the SNFs‐assisted exfoliation, the exfoliated BNNSs were kept in the supernatant of the SNFs dispersion, and the hybrid dispersion of SNFs and BNNSs (S_n_‐BNNSs, n represented for the initial SNFs concentration) could be obtained by centrifugation (Figure S7, Supporting Information). In addition, the S_n_‐BNNSs dispersion could be produced in a large scale through the SNFs‐assisted exfoliation process (Figure 2b). Stable aqueous dispersions with higher net concentrations of BNNSs were obtained by optimizing the initial SNFs concentration (Figure 2c; Figure S8, and Table S2, Supporting Information). When the SNFs concentration increased from 0.25 to 1 mg mL^−1^, the net BNNSs concentration in the hybrid dispersion increased dramatically. Notably, the net BNNSs concentration (1.31 mg·mL^−1^) and its yield (26.2%) reached the maximum value at a SNFs concentration of 1 mg mL^−1^, which confirmed that the efficiency of our developed SNFs‐assisted exfoliation process was higher than those in the previously reported liquid‐phase exfoliation process (Table S3, Supporting Information). With a further increase of SNFs concentration, the net BNNSs concentrations in S_2_‐BNNSs and S_4_‐BNNSs dispersions decreased to 0.93 and 0.75 mg·mL^−1^, respectively. Nevertheless, the net BNNSs concentrations in S_2_‐BNNSs and S_4_‐BNNSs dispersions were significantly higher than that without the assistant of SNFs (0.41 mg mL^−1^). The SNFs concentration of 1 mg mL^−1^ was determined as the optimal exfoliation concentration to obtain the highest BNNSs yield, excessive SNFs concentrations increased the tendency of SNFs to re‐agglomerate into larger fiber sizes, thus reducing BNNSs exfoliation efficiency.^[^ ^24^ ^]^ Furthermore, the value of the net BNNSs concentration induced by per concentration of SNFs was determined as the exfoliation rate, and was plotted against the initial SNFs concentrations (Figure 2d). As the SNFs concentrations increased from 0 to 0.5 mg mL^−1^, an increase in exfoliation rate from 0 to 132% was observed. Different from the SNFs concentration at which the maximum net BNNSs concentration was obtained, the ability of SNFs to assist the exfoliation of h‐BN was decreased when the SNFs concentration was higher than 0.5 mg mL^−1^. The lowest SNFs concentration of 0.5 mg mL^−1^ achieved the highest exfoliation rate of BNNSs, which might be attributed to the fact that SNFs rarely aggregated at lower concentrations and were well dispersed in the aqueous phase, facilitating the BNNSs exfoliation.^[^ ^24^ ^]^ These results above implied that SNFs had an excellent ability to assist the exfoliation of h‐BN at a very low optimal SNFs concentration (0.5 mg mL^−1^). Considering that dispersion property in the aqueous phase was a critical characteristic to affect the application of S_n_‐BNNSs in the fabrication of uniform nanocomposite membranes, the dispersions of U‐BNNSs (exfoliated BNNSs without SNFs assistance) and S_1_‐BNNSs with the maximum net BNNSs concentration were further evaluated using *ζ*‐potential measurement. The S_1_‐BNNSs dispersion showed a higher absolute value of zeta potential (−28.7 mV) compared with that of U‐BNNSs (−21.5 mV) in neutral aqueous solution. The higher zeta potential indicated that the S_1_‐BNNSs could be dispersed more stably in water. After being stored for 20 days at room temperature, the S_1_‐BNNSs dispersion only showed a 27.9% decrease in original concentration (2.21 mg mL^−1^), whereas U‐BNNSs dispersion decreased by 70.7%, confirming that SNFs have excellent ability to disperse the exfoliated BNNSs in aqueous phase.

**Figure 3:**
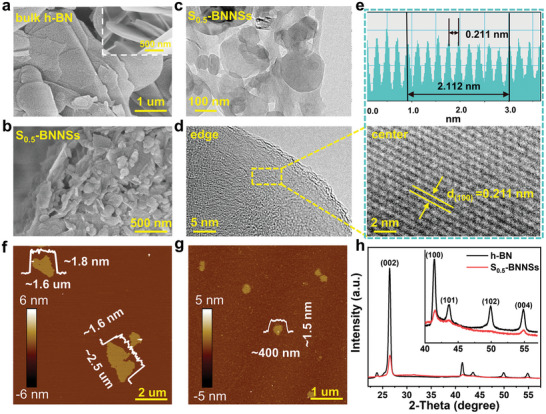
SEM images of a) h‐BN (Inset shown partial enlargement) and b) S_0.5_‐BNNSs. c) Low‐magnification TEM image and d) HR‐TEM image of S_0.5_‐BNNSs. e) Partial enlargement HR‐TEM image of S_0.5_‐BNNSs (bottom) and the corresponding lattice fringes profile (up). f,g) Selected AFM images height profiles of S_0.5_‐BNNSs, the insets were typical height profiles of the selected nanosheets. h) powder XRD patterns, inset was an enlargement of the 40–53°.

To examine the morphological change of h‐BN after the SNFs‐assisted exfoliation, SEM images of the pristine h‐BN and S_0.5_‐BNNSs were collected (**Figure** **3**a,b). Compared with the bulk h‐BN precursors (Figure 3a), the S_0.5_‐BNNSs demonstrated a much smaller size and abundant fracture edges with a fluffy and nanosheet‐like morphology (Figure 3b). Moreover, TEM image clearly suggested that the S_0.5_‐BNNSs had an ultra‐thin, transparent and overlapping planar structure with abundant edges formed during SNF‐assisted exfoliation (Figure 3c). As characterized by high‐resolution TEM, the edge area of S_0.5_‐BNNSs exhibited less ordered and a few defects (Figure 3d), which could be ascribed to the sonicating‐induced fracture and hydrolysis of defective sites.^[^ ^25^ ^]^ Meanwhile, the well‐ordered lattice structure remained intact without defective holes or dislocations on the basal plane, exhibiting clear h‐BN crystal lattice fringes with d‐spacing of 0.221 nm (Figure 3e), which was close to the (100) interplanar spacing of h‐BNNSs (0.217 nm, JCPDS card no. 34–0421). This result indicated that the S_0.5_‐BNNSs still retained highly intact crystalline structure after SNFs‐assisted exfoliation. The ultra‐thin feature of S_0.5_‐BNNSs could be further confirmed by AFM examinations (Figure 3f,g). Small smooth particles with a uniform thickness between 1 to 2 nm and a rang in lateral size from the micrometer to nanometer scales were observed for the S_0.5_‐BNNSs. Owing to that the thickness of monolayered BNNS was ≈0.4–0.5 nm,^[^ ^26^ ^]^ the S_0.5_‐BNNSs were consisted of ≈2–5 atomic layers. Besides, XRD patterns showed that the crystalline structure was well preserved after the SNFs‐assisted exfoliation of h‐BN (Figure 3h). In the XRD patterns, two typical characteristic diffraction peaks were observed for S_0.5_‐BNNSs at 26.4° and 42.8°, corresponding to the (002) and (100) planes of h‐BN, respectively,^[^ ^27^ ^]^ which indicated that few basal‐plane defects were produced in S_0.5_‐BNNSs during SNFs‐assisted exfoliation. The decrease in the intensity of these diffraction peaks for S_0.5_‐BNNSs suggested a decrease in the lateral size after the exfoliation process. These results above further confirmed that the developed SNFs‐assisted exfoliation method could effectively peel bulk h‐BN into ultrathin few‐layer nanosheets with few defects.

**Figure 4:**
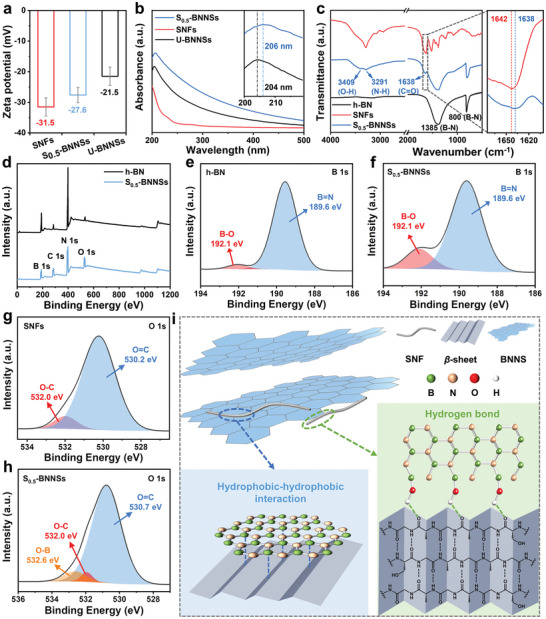
a) Zeta potential and b) UV–vis spectra of U‐BNNSs, SNFs, and S_0.5_‐BNNSs aqueous dispersions (Inset was an enlargement of the 200–220 nm). c) FT‐IR spectra of h‐BN, SNFs, and S_0.5_‐BNNSs. d) XPS survey of the h‐BN and S_0.5_‐BNNSs, e,f) high‐resolution XPS spectra of B 1s and g,h) O 1s of h‐BN and S_0.5_‐BNNSs. i) The interactions between SNFs and BNNSs during the SNFs‐assisted liquid exfoliation. Data presented as mean ± SD, n = 3.

To investigate the deep insights on the interfacial interaction between the BNNSs and SNFs, several characterization techniques, including zeta potential, (UV–vis) spectrophotometry, FT‐IR, and X‐ray photoelectron spectroscopy (XPS), were employed. Through *ζ*‐potential analysis (**Figure** **4**a), SNFs (−31.5 mV), U‐BNNSs (−21.5 mV), and S_0.5_‐BNNSs (−27.6 mV) exhibited negative surface charges in the neutral aqueous dispersions, demonstrating that electrostatic repulsion contributed to the formation of stable S_n_‐BNNSs dispersions. Although U‐BNNSs dispersion had a zeta potential of −21.5 mV, it could not be well dispersed in water due to its hydrophobicity. Moreover, the UV–vis absorption peak for the S_0.5_‐BNNSs dispersion showed a redshift from 204 (for the U‐BNNSs dispersion) to 206 nm (Figure 4b), which might originate from the strong physical absorption between hydrophobic planes of BNNSs and hydrophobic *β*‐sheet secondary structure of SNFs, which was consistent with the similar results for cellulose nanofibers/BN nanotubes.^[^ ^23^, ^28^ ^]^ In the FT‐IR spectra (Figure 4c), the peaks of S_0.5_‐BNNSs at 800 and 1385 cm^−1^ were assigned to the B─N─B bending vibration and B─N stretching vibration, respectively,^[^ ^27^ ^]^ indicating that the covalent bond between the B and N atoms of h‐BN was not ruptured after SNFs‐assisted exfoliation. The large area of the O─H stretching vibrational peak was observed at 3409 cm^−1^, and multiple peaks in the region of 1200–900 cm^−1^ were presumed to be B─O group peak, which were attributed to the sonication‐assisted hydrolysis of defective sites during the exfoliation.^[^ ^25^, ^27^, ^29^ ^]^ These hydrolyzed defective sites might provide sites for the formation of hydrogen bonding interactions between BNNSs and SNFs. The FT‐IR spectrum of the SNFs had a strong carbonyl stretching absorption at ≈1642 cm^−1^,^[^ ^30^ ^]^ which was slightly shifted to 1638 cm^−1^ in the FT‐IR spectrum of S_0.5_‐BNNSs. This could be caused by the hydrogen‐bonding interaction between carbonyl groups on the SNFs and the hydroxyl groups on BNNSs. More importantly, XPS was conducted to determine the hydrogen bonding interactions between the SNFs and BNNSs (Figure 4d–h). From the XPS spectra of B 1s in h‐BN (Figure 4e) and S_0.5_‐BNNSs (Figure 4f), more B atoms bound to O atoms were observed from the peak intensity at 192.1 eV. Meanwhile, a new peak at 532.6 eV (O─B) was observed in the XPS spectra of O 1s for S_0.5_‐BNNSs (Figure 4h),^[^ ^31^ ^]^ further confirming that the hydroxyl groups could be introduced to the edges of the fractured BNNSs due to the combination of intense mechanical shearing, ultrasonic cavitation, and solvent polarity effect.^[^ ^32^ ^]^ Besides, the pristine SNFs exhibited a dominant XPS peak at 530.2 eV, attributing to amide groups (N─C═O).^[^ ^16c^ ^]^ Compared with SNFs, the O 1s binding energy of the C═O groups in S_0.5_‐BNNSs obviously shifted to higher value (530.7 eV), while the O 1s of the C─O groups (532.0 eV) remained almost unchanged (Figure 4g,h). The higher binding energy should be resulted from the formed hydrogen bonds between the O atom in SNFs and the H atom in BNNSs, which decreased the electron density around O atoms. Thus, the changes of O 1s binding energy in the S_0.5_‐BNNSs confirmed the formation of hydrogen bonding between the C═O groups of SNFs and the B─OH groups of BNNSs. From the characterizations above, hydrophobic to hydrophobic interactions and hydrogen bonds could be formed between SNFs and the fractured h‐BN. Subsequently, the fractured bulk h‐BN was further expanded and exfoliated to form BNNSs by sonication in the SNFs dispersion (Figure 4i). At the same time, the SNFs dispersion could improve the dispersibility of exfoliated BNNSs in the aqueous phase by capturing the exfoliated BNNSs through the two interactions mentioned above and preventing the reaggregation of BNNSs among themselves and with the fractured BN, therefore, the formation of stable SNF mixed dispersions.^[^ ^33^ ^]^

Delighted by the good performance of DESs‐exfoliated SNFs on exfoliation of h‐BN in the aqueous phase, the universality of this SNFs‐assisted exfoliation method was evaluated on other 2D‐layered materials, i.e., molybdenum disulfide (MoS_2_), tungsten sulfide (WS_2_), and graphite. With the same experimental steps and optimal parameters (SNFs concentration of 1 mg·mL^−1^) used in h‐BN exfoliation, the exfoliated nanosheets of MoS_2_ and WS_2_ could be well dispersed in SNFs/nanosheets hybrid dispersions at high net nanosheet concentrations of 1.28 and 1.06 mg·mL^−1^, which were significantly higher than that of corresponding nanosheets concentrations (0.45 and 0.38 mg·mL^−1^, respectively) without the assistant of SNFs (Figure S9a,b, Supporting Information). Meanwhile, the nanosheet yields in the SNFs‐assisted process could reach up to 25.6% and 21.2%, respectively (Table S4, Supporting Information). In addition, SEM images showed that the bulk MoS_2_ and WS_2_ crystal with dimensions of several micrometers fractured into nanoscale fragments, and the ultrathin feature of nanosheets were confirmed by the AFM images (Figure S9c–h, Supporting Information). Unfortunately, graphite nanosheets were hardly to be exfoliated by this proposed SNFs‐assisted exfoliation process (Figure S10, Supporting Information). The poor exfoliation behavior of graphite was probably caused by the weak fragmentation effect during sonication. Thus, un‐fractured and thick bulk crystals were not favorable for subsequent exfoliation and dispersion.^[^ ^34^ ^]^ In summary, scalable DESs exfoliation of silk fibers efficiently prepared SNF with a controllable fibrillar structures (contour lengths of 0.1–2.6 µm and diameters of 18–100 nm) and intact crystalline features, successfully preserving the hierarchical structure of natural silk fibers. In subsequent applications, the developed SNFs‐assisted exfoliation method could effectively peel bulk h‐BN into ultrathin few‐layer nanosheets with few defects by hydrophobic‐hydrophobic and hydrogen bonds interactions.

**Figure 5:**
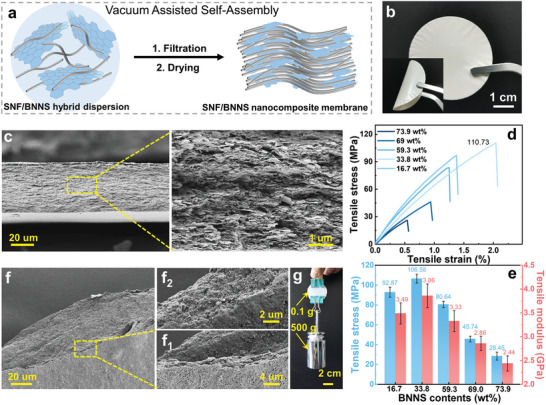
a) The fabrication process of SNFs/BNNSs nanocomposite membranes. b) Photograph of the SNFs/BNNSs‐59.3% membrane, c) cross‐sectional SEM images of SNFs/BNNSs‐59.3% membrane. d) Tensile stress‐strain curves, e) tensile strength and tensile modulus of the SNFs/BNNSs membranes with different BNNS contents. f) SEM images of the fracture of f_1_) SNFs/BNNSs‐59.3% and f_2_) SNFs/BNNSs‐73.9% membranes after the tensile test. g) Lifting a 500 g weight by SNFs/BNNSs‐59.3% membrane of 0.1 g. Data presented as mean ± SD, n = 3.

SNFs have been successfully applied to the basic components of materials, including 2D composite membranes and 3D aerogels.^[^ ^2^, ^35^ ^]^ For the practical SNFs‐based thermal management composite membrane application, mixed dimensional SNFs/BNNSs nanocomposite membranes were directly fabricated by vacuum filtration of S_n_‐BNNSs hybrid dispersions (**Figure** **5**a). The mass fractions of BNNSs in the obtained SNFs/BNNSs nanocomposite membranes were 73.9%, 69.0%, 59.3%, 33.8%, and 16.7%, respectively, based on net BNNSs concentrations in S_n_‐BNNSs hybrid dispersions (Table S2, Supporting Information). The obtained membranes were labeled as SNFs/BNNSs‐73.9%, SNFs/BNNSs‐69.0%, SNFs/BNNSs‐59.3%, SNFs/BNNSs‐33.8%, and SNFs/BNNSs‐16.7%, respectively. Moreover, a tight nanocomposite membrane without splitting could be obtained owing to the excellent stability of the BNNSs in SNFs dispersion (Figure 5b). Through morphology and dimension characterizations, the SNFs have a contour length of 0.1–2.6 µm and a diameter of 18–100 nm and the BNNSs have a uniform thickness between 1–2 nm and a nanoscale lateral size. In subsequent vacuum‐assisted filtration, attributed to the hydrophobic to hydrophobic and hydrogen‐bonding interactions, the SNFs network could easily capture the exfoliated BNNSs that enabled the BNNSs to spontaneously and uniformly occupy vacant space in the SNFs network without significant aggregation^[^ ^36^ ^]^ (Figure S11, Supporting Information). The cross‐sectional SEM images of nanocomposite membrane showed that the BNNSs were compactly stacked layer‐by‐layer in the SNFs matrix to form a lamellar structure (Figure 5c), which was beneficial for anisotropic thermal conductivity and heat transfer along the in‐plane direction.^[^ ^37^ ^]^ Considering that these membranes would potentially suffer from large deformations in practical applications, the stretchability of these SNFs/BNNSs nanocomposite membranes with different BNNSs loadings was determined (Figure 5d,e). Due to the reinforcing effect of the SNFs network and the strong interaction between SNFs and BNNSs (hydrophobic–hydrophobic and hydrogen bond interactions), the mechanical properties of nanocomposite membrane increased gradually when the amount of SNFs increased from 16.7% to 33.8%. However, the lamellar structure was deteriorated by the aggregation of BNNSs at higher contents (more than 33.8%), leading to the decrease of their mechanical properties. The main reason was that the introduction of excess BNNSs inevitably destroyed the continuity of the SNFs network and lead to the aggregation of BNNSs from hydrophobic–hydrophobic interactions, causing it to generate stress concentration points when it was stressed, which decreased its tensile strength. As shown in Figure 5d,e, the optimal mechanical property was achieved at the SNFs/BNNSs‐33.8%, including a tensile strength of 110.73 MPa, a tensile modulus of 3.86 GPa (Figure 5e) and a toughness of 1295.4 KJ·m^−3^ (Figure S12, Supporting Information). The micromorphology of the fractured cross‐section of the SNFs/BNNSs nanocomposite membrane has been investigated to understand the mechanism of robust mechanical properties (Figure 5f), which exhibited an overall stepped‐like laminar structure. In the case of SNFs/BNNSs‐59.3% (Figure 5f_1_), BNNSs were well‐embedded in the SNFs network, resulting in a pattern of alternating stacks of BNNSs and SNFs, which was conducive to the formation of stronger interlayer interactions. However, for SNFs/BNNSs‐73.9% (Figure 5f_2_), bare SNFs were hardly visible and BNNSs were slipped and pulled out. In this case, a large amount of BNNSs resulted in severe aggregation of the nanosheets in the nanocomposite membrane, which could explain the poor mechanical performance of SNFs/BNNSs‐73.9%.^[^ ^38^ ^]^ Meanwhile, a small size (100 mg) of SNFs/BNNSs‐59.3% membrane could easily lift the weight of more than 5000 times its own weight (Figure 5g). These comparative results indicated that synergistic effects of nanofibers and nanosheets of forming the laminate structure endowed the membrane with robust mechanical properties.

**Figure 6:**
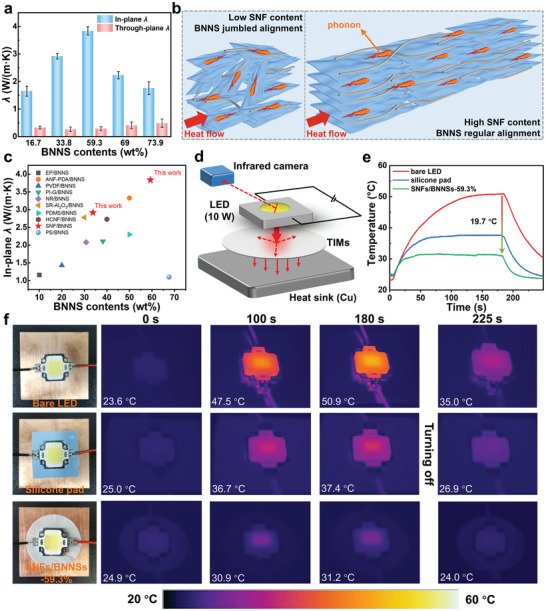
a) Thermal conductivity coefficients of the SNFs/BNNSs nanocomposite membranes at room temperature. b) Mechanism schematic diagram for thermal conduction in SNFs/BNNSs‐73.9% membrane (left) and SNFs/BNNSs‐59.3% membrane (right). c) Comparison of the in‐plane *λ* of SNFs/BNNSs membranes and other published work about polymer‐based BNNS nanocomposites (Table S5, Supporting Information). d) Schematic diagram for heat dissipation test system for high‐power LED modules. e) Surface temperature‐time curves of the LED modules using air, commercial silicone pad and SNFs/BNNSs‐59.3% membrane to dissipate heat, respectively, and f) the corresponding infrared thermal images.

Thermal conductivity coefficients (*λ*) of the developed SNFs/BNNSs nanocomposites with different BNNS loadings was finally evaluated (**Figure** **6**a). Benefiting from the dense laminated structure in the SNFs/BNNSs nanocomposite membranes, BNNSs were structurally aligned in the in‐plane direction of the SNFs network, which could maximize the in‐plane thermal conductivity and preserve a low through‐plane thermal conductivity.^[^ ^37^, ^39^ ^]^ The in‐plane *λ* of the nanocomposites increased with the increase of the BNNSs content initially and then decreased (Figure 6a), and reached the highest value of 3.84 W·m^−1^·K^−1^ in the SNFs/BNNSs‐59.3%. This highest heat‐dissipating efficiency of the SNFs/BNNSs‐59.3% membrane probably originated from its much higher in‐plane λ than the through‐plane *λ*. The improved in‐plane *λ* was mainly related to the continuous thermal conductive channels formed by the strong interactions between SNFs and BNNSs and the significant reduction of the phonons scattering owing to the high *β*‐sheet content and crystallinity of DESs‐exfoliated SNFs.^[^ ^40^ ^]^ An obviously decrease in the in‐plane *λ* occurred at high BNNS contents because the aggregation of BNNSs resulted in the increase in thermal resistance. In the thermally conductive SNFs/BNNSs nanocomposite membranes, the BNNSs were firmly captured by the SNFs through hydrophobic‐hydrophobic and hydrogen bond interactions, which resulted in a highly aligned orderly along the in‐plane direction of the membranes, realizing efficient formation of numerous and continuous thermal conductive pathways at the in‐plane of nanocomposite under the low loading of BNNSs.^[^ ^37^, ^41^ ^]^ Consequently, the heat transfer was increased, and the thermal conductivities of the SNFs/BNNSs nanocomposite membranes were effectively improved. In contrast, the excess BNNSs were not well dispersed by SNFs and were randomly distributed and partially agglomerated in the nanocomposite membranes with too high BNNSs content, which cannot form continuous BNNS‐BNNS thermal conductive pathways and thus the phonon was scattered and disordered (Figure 6b).^[^ ^42^ ^]^ Moreover, the thermal conductivity of the SNFs/BNNSs‐59.3% membrane was higher than those of the previously reported polymer‐based BNNSs nanocomposites (Figure 6c; Table S5, Supporting Information), confirming the superiority of our developed SNFs‐based nanocomposite for boosting the thermal conductivity.

Generally, the rapid increase in power density of electronic devices would result in more serious heat dissipation problems. The thermal interface materials (TIMs) between the heat source and heat sink were the key medium to transfer the generated heat, which had extensively used to dissipate heat in electronic devices.^[^ ^38^, ^43^ ^]^ Owing to the excellent mechanical properties and superior in‐plane *λ*, the SNFs/BNNSs nanocomposite membranes could be potentially applied as a flexible TIM for cooling high‐power density. In order to preliminarily evaluate the actual heat dissipation performance of SNFs/BNNSs nanocomposites, which were utilized as the TIMs between the operating high‐power LED modules (10 W) and the heat sink, the infrared images and surface temperatures were captured simultaneously by an IR camera (Figure 6d–f). With an irradiation time of 180 s, the equilibrium temperature of the LED equipped with SNFs/BNNS‐59.3% membrane only rose slowly from 24.9 to 31.2 °C, which was much lower than the bare LED (50.9 °C) and the one attached with commercial silicone pad (3 W·m^−1^·K^−1^, 37.4 °C). After turning off the LED, the temperature of SNFs/BNNS‐59.3% membrane‐attached LED dropped to 24.0 °C in 45 s, while the commercial silicone pad‐attached and bare LEDs only dropped to 26.9 and 35.0 °C, respectively. These results above strongly confirmed the superior heat‐dissipating efficiency of SNFs/BNNSs nanocomposite membrane in comparison with the commercial silicone pad (3 W·m^−1^·K^−1^), and verified the great potential of SNFs/BNNSs nanocomposite membrane for high‐temperature thermal management in electronic devices.

## 3 Conclusion

In conclusion, a facile, high‐efficiency, scalable and environmentally friendly top‐down strategy was developed to direct exfoliation of silk fibers to generate SNFs by using amino acids/D‐sorbitol DESs. The DESs‐exfoliated SNFs had controllable contour lengths of 0.1–2.6 µm and diameters of 18–100 nm, preserving the natural hierarchical stuctures such as fibrillar structure and crystalline features. As an important application, the DESs‐exfoliated SNFs could efficiently assist the exfoliation of several 2D‐layered materials, i.e., h‐BN, MoS_2_, and WS_2_, to form the corresponding nanosheets. 1 mg·mL^−1^ was observed to be the optimized SNFs concentration to exfoliate the 2D‐layered materials, and the concentrations of the h‐BN, MoS_2_, and WS_2_ nanosheets could reach up to 1.31, 1.28, and 1.06 mg·mL^−1^, respectively. More importantly, the nanocomposite membranes manufactured from the SNFs‐assisted dispersion of BNNSs with a content of 59.3% showed excellent mechanical properties (tensile strength of 416.7 MPa, tensile modulus of 3.86 GPa and toughness of 1295.4 KJ·m^−3^) and thermal conductivity (in‐plane thermal conductivity coefficient of 3.84 W·m^−1^·K^−1^). By comparing with the reported thermal conductivity of the polymer‐based BNNSs nanocomposites, the DESs‐exfoliated SNFs showed superior thermal conductivity and were more promising renewable polymer substrates for thermally conductive 2D nanosheets. Besides, the constructed SNFs/BNNSs nanocomposites showed superior cooling efficiency compared with the commercial silicone pad. We believe that DESs can be a highly promising solvent to exfoliate biopolymers to produce biopolymer nanofibers, and the generated biopolymer nanofibers with amphiphilicity can be used as the renewable additive to assist the generation of 2D‐layered material nanosheets.

## 4 Experimental Section

All the experimental details are reported in Supporting Information.

## Conflict of Interest

The authors declare no conflict of interest.

## Author Contributions

Y.W. performed all experiments, collected, and analyzed data, wrote and revised manuscript, assisted in the experimental design and ideas conceptualization. Z.Y. conducted the formal analysis. B.J. assisted in the DESs preparation and SNFs exfoliation. L.C. collected the references. C.Y. assisted in the presentation of conceptualization. Z.X. and T.M. conceived the idea and designed the experiments. Z.X. and F.P. provided the project administration and supervision. Z.X. reviewed the original and revised manuscripts. All authors discussed the results and edited the manuscript.

## Supporting information

Supporting Information

## Data Availability

The data that support the findings of this study are available from the corresponding author upon reasonable request.
