# Supplementary material for: Natural Deep Eutectic Solvent‐Assisted Construction of Silk Nanofibrils/Boron Nitride Nanosheets Membranes with Enhanced Heat‐Dissipating Efficiency
Source: Adv Sci (Weinh). 2024 Jul 25;11(36):2403724. doi: 10.1002/advs.202403724 (PMC11529046; doi:10.1002/advs.202403724)
Supplement: Supplementary file 1 — Supporting Information [file ADVS-11-2403724-s001.docx]

**Supporting Information for:**

**Natural Deep Eutectic Solvent-Assisted Construction of Silk Nanofibrils/Boron Nitride Nanosheets Membranes with Enhanced Heat-Dissipating Efficiency**

Yang Wang, Zhaohui Yang, Bingzheng Jia, Lan Chen, Chuanyu Yan, Feng Peng*, Tiancheng Mu, and Zhimin Xue*

**Experimental Section**

**Chemicals and Materials**

Sodium carbonate (98%), D-Sorbitol (99%), L-Glutamic acid (99%) and L-Proline (98%) were purchased from Shanghai Aladdin Biochemical Technology Co., Ltd. (Shanghai, China). The raw silkworm cocoon chips were collected Zhejiang province in China. Hexagon boron nitride (h-BN, ~1-3 μm average lateral size), molybdenum disulfide (MoS_2_), tungsten sulfide (WS_2_), and graphite were purchased from Macklin Chemical Co., Ltd. (Shanghai, China).

**DESs-assisted exfoliation of silk fibers**

Silkworm cocoon chips were degummed in the boiling Na_2_CO_3_ aqueous solution (0.5 wt%) for 0.5 h, then thoroughly washed in deionized water and dried at 60 ^o^C for 12 h. L-Glutamic acid/D-Sorbitol (GS) and L-Proline/D-Sorbitol (PS) DESs were prepared by mixing D-Sorbitol with L-Glutamic acid or L-Proline in the molar ratio of 2:1 at 130 ^o^C until the transparent and homogeneous liquid were obtained. For the liquid exfoliation of silk fibers, the degummed cocoon silk fibers were immersed into DESs with a weight ratio of 1:100 and stirred vigorously at 100 ^o^C with a stirring paddle at 500 rpm. After 20 hours, deionized water was added to dilute the viscous pulp-like mixture. The diluted mixture was then separated by filtration and washed with deionized water to remove the DESs until the filtrate was neutral, followed by lyophilization of the solid residue to obtain DES-exfoliated silk fibers. The DESs-exfoliated silk fibers were added to water with a weight ratio of 1:500 and stirred vigorously at 500 rpm on a magnetic stirrer to form silk fiber suspensions. To generate higher yields of SNFs, the silk fiber suspension was sonicated at ambient temperature in a KQ-400DE ultrasonic machine (Ningbo Scientz Biotechnology) at 400 W for 4 h. Finally, the exfoliated SNFs dispersions were achieved by centrifugation twice at 2000, 5000 and 8000 rpm for 30 min, respectively. The SNFs dispersions were marked as DESs-SNFs-X, where X was the centrifugation speed.

**Preparation of BNNSs by the SNFs-assisted liquid exfoliation**

In a typical SNFs-assisted exfoliation process, h-BN (5 mg mL^−1^) was mixed in 500 mL SNFs dispersion in different concentrations and stirred vigorously at room temperature for 30 min. Then, the mixed suspensions were sonicated at ambient temperature in a KQ-400DE ultrasonic bath at 400 W for 8 h. The resulted dispersions were centrifuged twice at 2000 rpm for 30 min each time to remove unexfoliated h-BN particles. The collected supernatant was the SNFs/BNNSs hybrid dispersions by SNFs-assisted exfoliation and stabilization, named “S_n_-BNNSs”, where n represented the initial SNF concentration. In control experiments, the unassisted BNNSs were fabricated using the same stirring and sonication process without SNFs, named “U-BNNSs”.

The concentrations of the SNFs/2D nanomaterial hybrid dispersions and net 2D nanomaterial concentration in hybrid dispersions were measured by the following method. The exfoliated S_n_-BNNSs dispersion was used as a sample. 10 mL of the exfoliated S_n_-BNNSs dispersion was placed into an oven at a temperature of 105 ^o^C until the dispersion was dried completely. The following equation relates C_Sn-BNNSs_, the concentration of the exfoliated S_n_-BNNSs dispersion (mg mL^−1^) to m, the dry weight of the exfoliated S-BNNSs dispersion (mg):

$$\text{C}_{\text{Sn-BNNS}}=\frac{\text{m}}{\text{10}}$$

The weight ratio of the SNFs in the exfoliated S_n_-BNNSs dispersion was detected by thermogravimetric analysis (TG) using the following formula:

$$\text{W}_{\text{SNFs}}\text{=}\frac{\text{WL}_{\text{Sn-BNNSs}}\text{-}\text{WL}_{\text{h-BN}}}{\text{WL}_{\text{SNFs}}\text{-}\text{WL}_{\text{h-BN}}}\times100\%$$

where W_SNFs_ is the weight ratio of the SNFs in the exfoliated S_n_-BNNSs dispersion, and WL_Sn-BNNSs_, WL_h-BN_, and WL_SNFs_ are the weight losses for the S_n_-BNNSs, the h-BN, and the SNFs, respectively. These weight losses were obtained based on the TG curve at 700 ^o^C. The TG curve of h-BN, SNFs, and S_n_-BNNSs were shown in **Figure S7**. The net BNNSs concentration (C_net-BNNSs_) in the exfoliated S_n_-BNNSs dispersion can be expressed by:

$$\text{C}_{\text{net-BNNSs}}\text{=}\text{C}_{\text{Sn-BNNSs}}\text{×}\left( \text{1-}\text{W}_{\text{SNFs}} \right)$$

Note that the net concentrations of other nanosheets in SNFs/nanosheets hybrid dispersions were also calculated using the same methodology as the net BNNSs concentrations, and the TG curve of different bulk 2D-crystals, SNFs, S_1_-MoS_2_NSs, S_1_-and WS_2_NSs were shown in **Table S4**.

**Fabrication of SNFs/BNNSs nanocomposite membranes**

The S_n_-BNNSs hybrid dispersions were used to fabricate SNFs/BNNSs nanocomposite membranes by vacuum-assisted filtration using the hydrophobic polyvinylidene difluoride filter membranes (pore size, 0.22 μm; diameter 50 mm), followed by drying at room temperature to obtain a series of membranes of about 0.1 g. The actual amount of BNNSs in the SNFs/BNNSs nanocomposite membranes obtained were 73.9, 69.0, 59.3, 33.8, and 16.7 wt%, respectively, according to the net BNNSs concentrations in S_n_-BNNSs, named “SNFs/BNNSs-X”, where X represented BNNSs mass ratio.

**Morphology and dimension characterizations**

The morphology of SNFs and S_n_-BNNSs were characterized by a scanning electron microscope (SEM, Hitachi Regulus8100, Japan), atomic force microscope (AFM, Bruker Multimode 8, USA), transmission electron microscope (TEM) and high-resolution transmission electron microscope (HRTEM) (Hitachi HT7800, Japan). For AFM experiments, samples were prepared by depositing the diluted dispersion (0.001 mg mL^−1^) onto a cleaved mica substrate and then dried in air. The tapping mode AFM imaging was used at a scanning range of 5 μm and a scanning speed of 1Hz. The AFM images were analyzed by the software NanoScopeAnalysis. For TEM and HRTEM experiments, a drop of the diluted dispersion (0.01 mg mL^−1^) was dropped on a carbon-coated Cu grid. The lattice spacing of BNNS was determined by the software Digital Micrograph (Gatan Software, Inc.).

**Structure characterizations**

The secondary structure of SNFs were examined by Fourier-transform infrared spectroscopy (FTIR) in the spectra region of 4000-400 cm^-1^ by accumulating 32 scans at a resolution of 4 cm^-1^. The content of secondary structure was performed by the software PeakFit V4.12 (SeaSolve Software, Inc.). The crystalline phase of the SNFs and S_n_-BNNSs were identified by X-ray diffractometer (Bruker D8 Advance, Germany) with a Cu Kα source from diffraction angles 2θ of 5-40° with a scan rate of 2° min^-1^. The degrees of the crystallinity for silks were estimated by analyzing the crystalline and amorphous components present in the azimuthal integration profiles.^[1]^ The degree of crystallinity was obtained using the software PeakFit V4.12 (SeaSolve Software, Inc.), in which the diffraction profile was deconvoluted.^[2]^ The relative crystallinity of the sample was calculated according to the following formular: crystallinity = (net area of diffraction peak/net area of diffraction peak + the area of the integrated intensity of the amorphous) ◊ 100%. The zeta potential of SNFs and S_n_-BNNSs in water were evaluated by a Zetasizer (zeta Litesizer 500, Anton Paar). The UV-vis spectra were measured using a UV-vis spectrophotometer (UH4150, Hitachi) at room temperature. The electron binding energies of B 1s and O 1s were analyzed by an X-ray photoelectron spectrometer (K-Alpha, Thermo Scientific) with a monochromatic Al-Kα X-ray source. Contact angle was measured in air atmosphere and room temperature by contact angle goniometer (SUNZERN SZ-CAMC32, China), to investigate the hydrophilic of DES-exfoliated SNFs. The thermal behaviors of the SNFs and S_n_-BNNSs were evaluated by thermogravimetic analysis (TGA, Netzsch TG 209 F3, Germany). Differential scanning calorimetry (DSC) measurements were carried out on a DSC 200 F3 from Netzsch Instruments. Each DESs sample was first cooled to -100 ^o^C at a rate of 5 ^o^C min^-1^ and then heated to 0 ^o^C at a rate of 5 ^o^C min^-1^.

**Characterization for the performance of SNFs/BNNSs nanocomposite membranes**

The mechanical properties of the samples were tested by a Shimadzu AG-X plus, loading rate was 5 mm min^-1^. In-plane thermal conductivity coefficient (*λ*_∥_) and through-plane thermal conductivity coefficient (*λ_⊥_*) were characterized with Hot Disk TPS 2500S thermal constant analyzer (AB Co., Sweden). Infrared thermal images were obtained by a Fotric 226 infrared thermal imaging camera (Fotric, China).

**Statistics Analysis**

Quantitative data was described in the form of means ± standard deviation, such as the error bars in the figures. At least three samples were conducted to test the average the secondary structures and crystallinity of SNFs, Zeta potential, mechanical properties, and in-plane thermal conductivity coefficients. All the data were analyzed statistically with Microsoft Office Excel (2016). The information about sample size was given in the respective figure captions.

**Supplementary Figures and Tables**


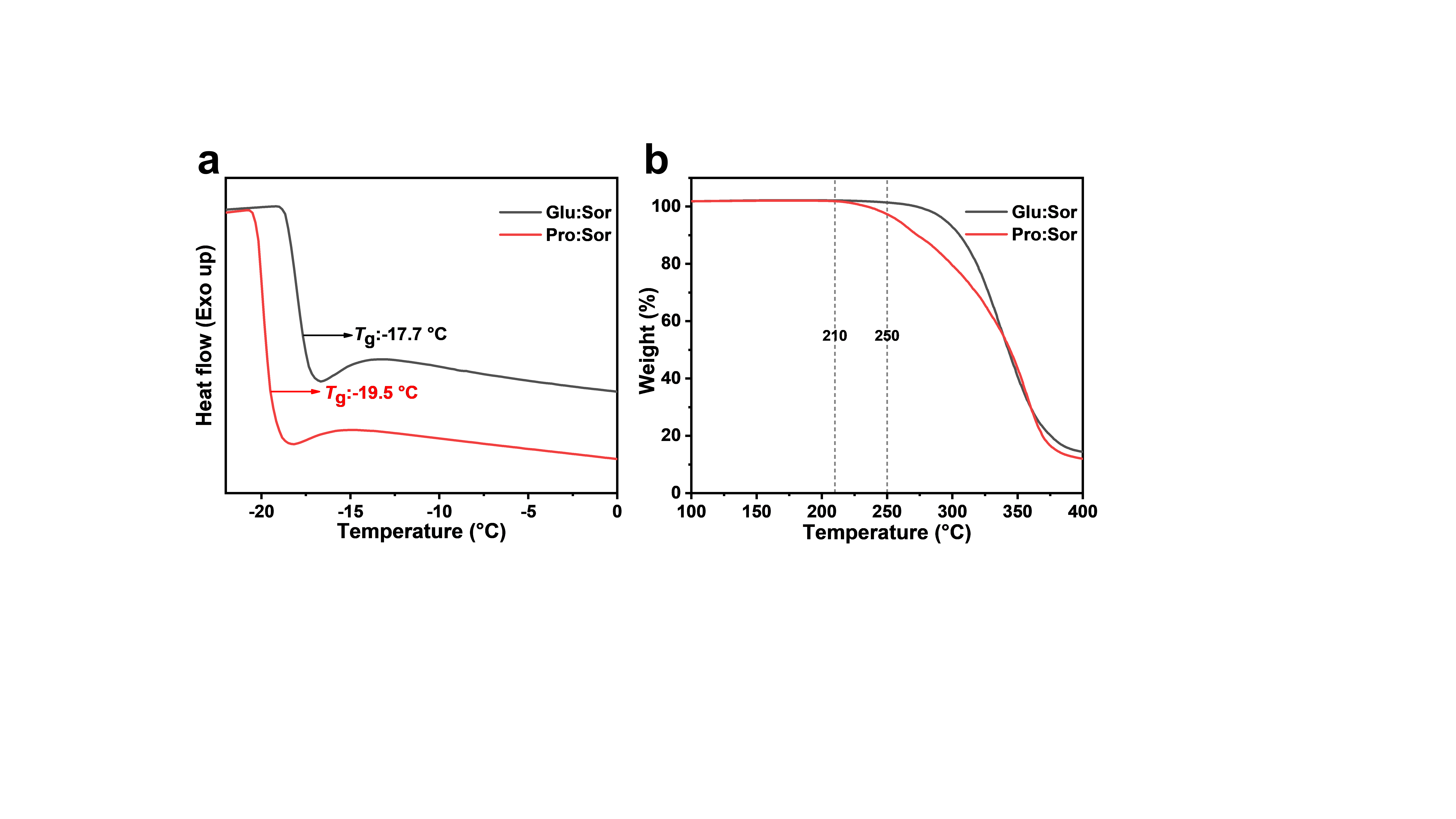


**Figure S1.** DSC curves (a) and TG curves (b) of L-Glutamic acid/D-Sorbitol (GS) and L-Proline/D-Sorbitol (PS) DESs.

**Table S1.** The effect of centrifugation rates on the concentration and yield of SNFs in the liquid exfoliation of silk fibers by different amino acid/sorbitol-based DESs.

| Sample | Centrifugation rates (rpm) | Concentration  (mg mL^−1^) | Yield (%) |
| --- | --- | --- | --- |
| GS-SNFs-2000 | 2000 | 1.15 | 57.5 |
| GS-SNFs-5000 | 5000 | 0.76 | 38.0 |
| GS-SNFs-8000 | 8000 | 0.41 | 20.5 |
| PS-SNFs-2000 | 2000 | 0.49 | 24.5 |
| PS-SNFs-5000 | 5000 | 0.31 | 15.5 |
| PS-SNFs-8000 | 8000 | 0.19 | 9.5 |


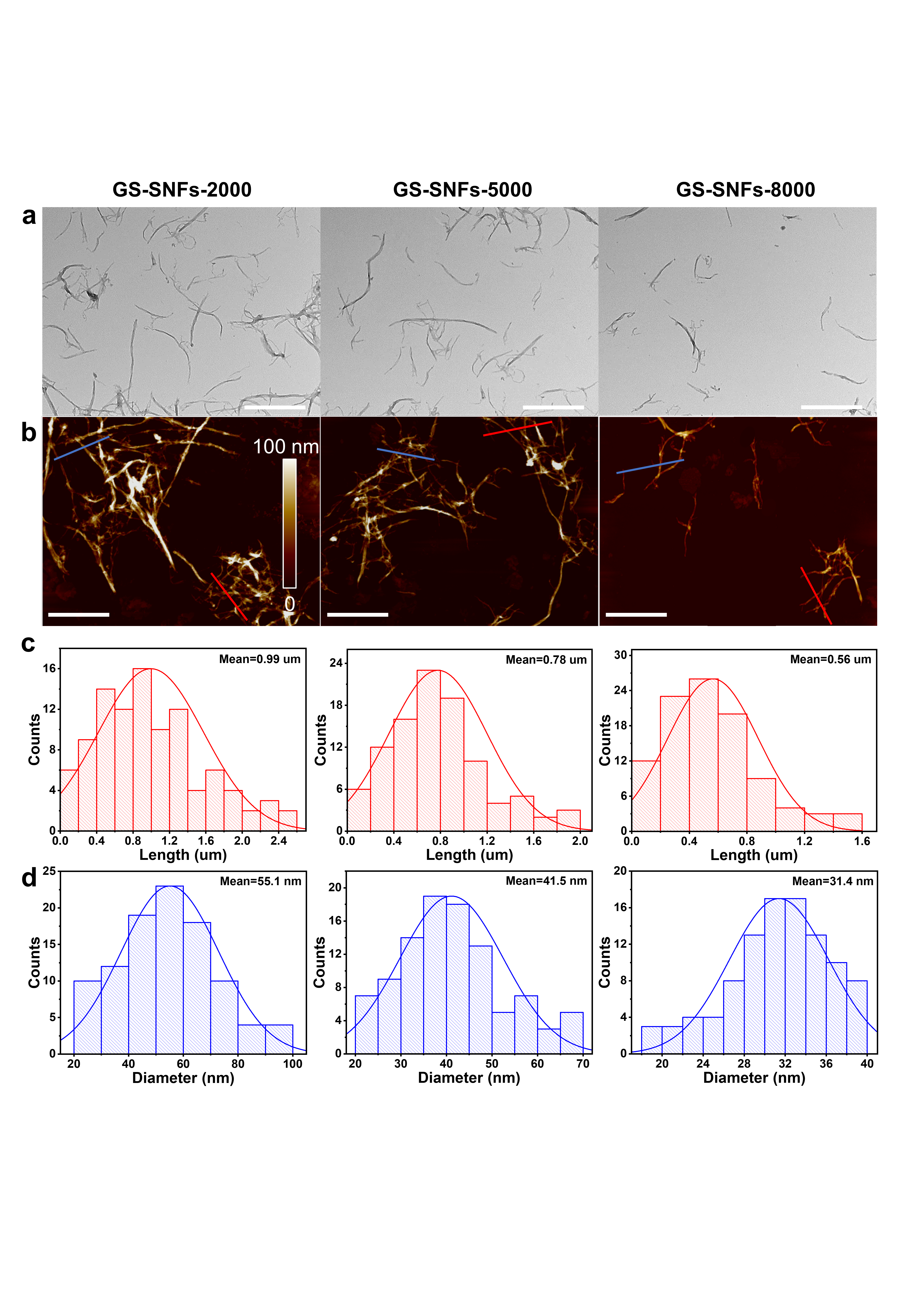


**Figure S2.** Characterization of morphology and size of GS-exfoliated SNFs. (a) TEM and (b) AFM images, (c) the length distribution and (d) the diameter distribution of SNFs from TEM images and AFM measurements respectively. Scale bars: 1 μm.


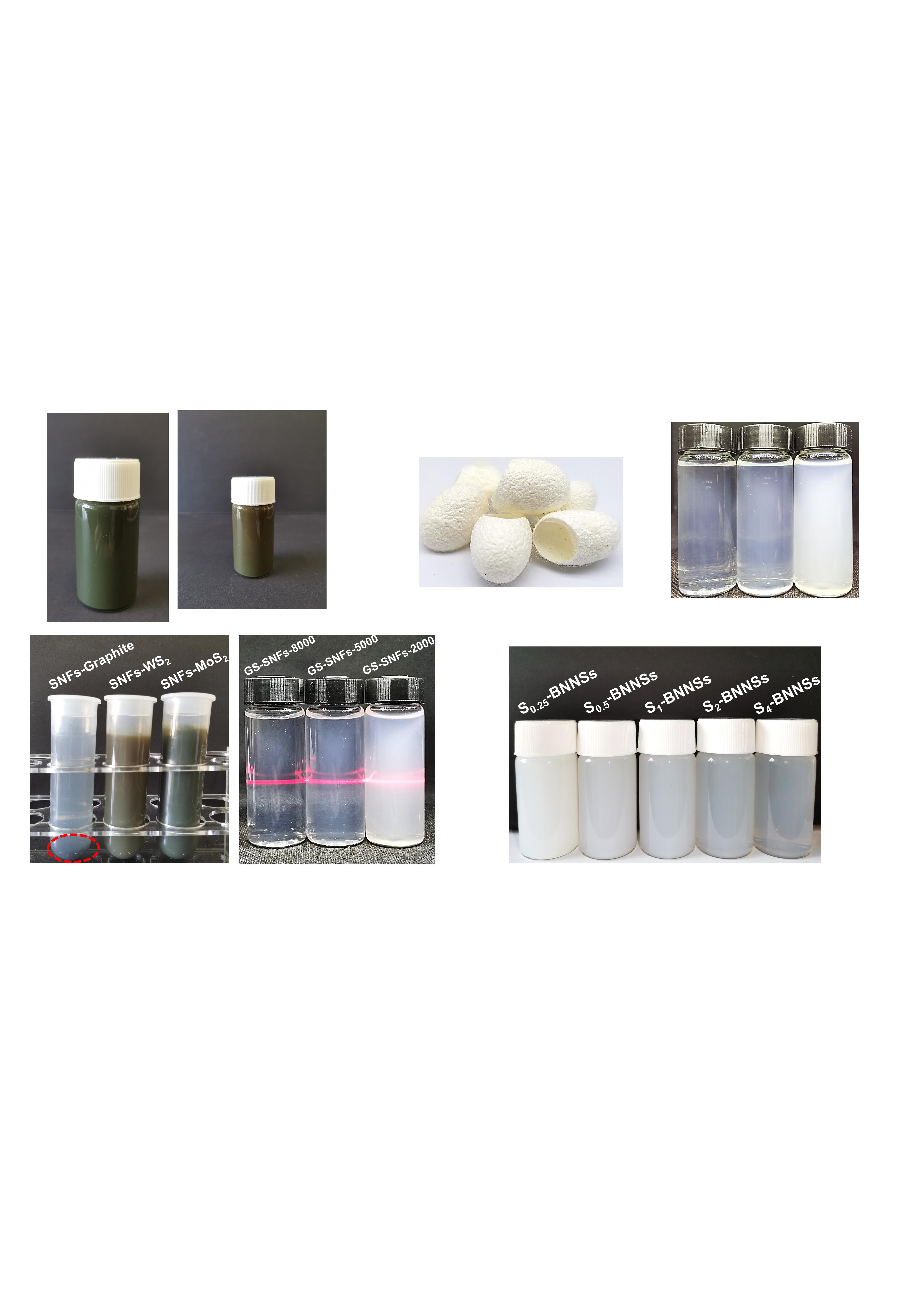


**Figure S3.** Photograph of DESs-exfoliated SNFs dispersions exhibiting strong Tyndall effect.


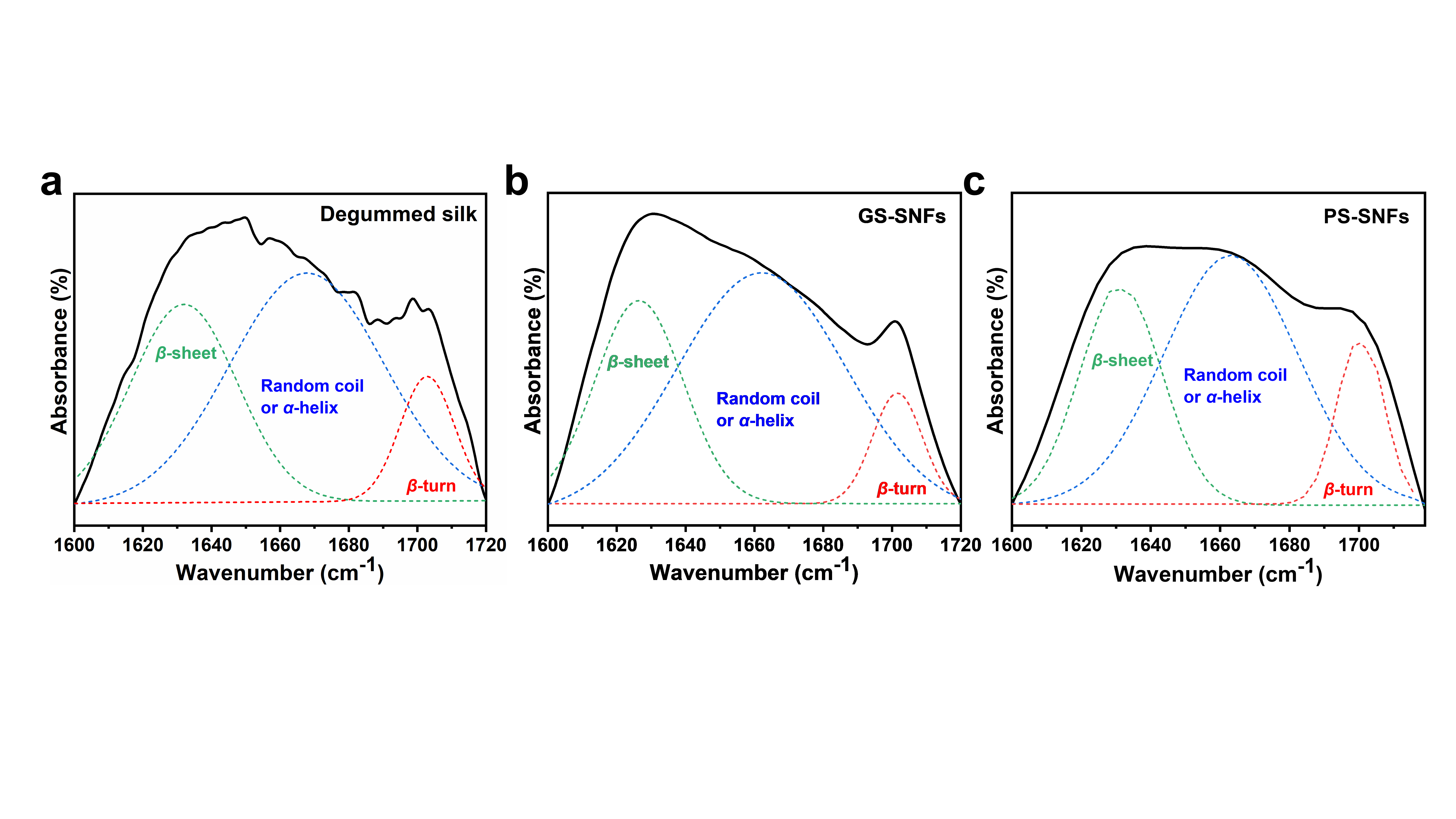


**Figure S4.** The deconvolution of FTIR spectra for the amide I band.


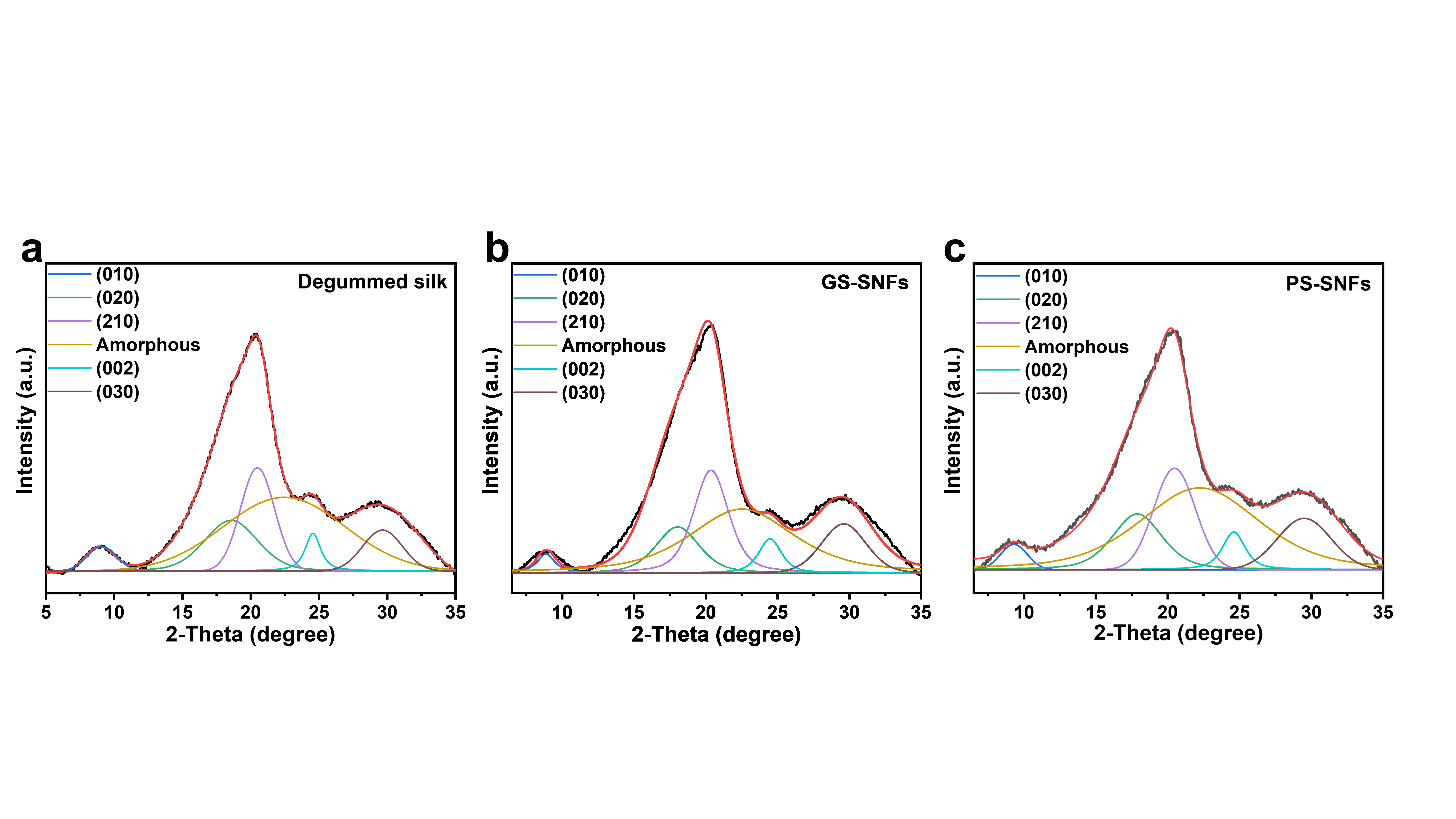


**Figure S5.** The deconvolution of XRD patterns.


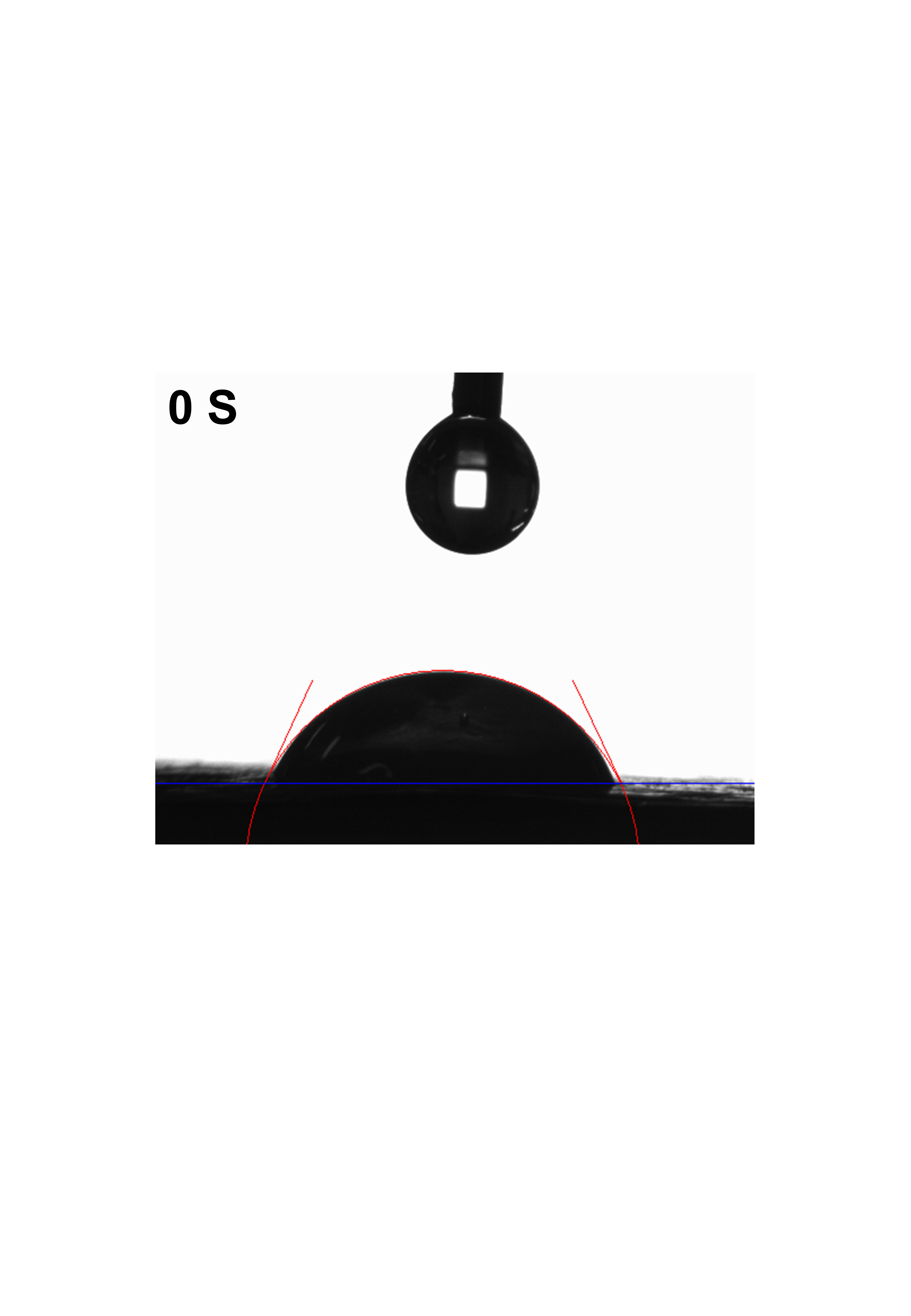


**Figure S6.** Analysis of the hydrophilic of surface of DESs-exfoliated SNFs membrane using a water contact angle experiment.


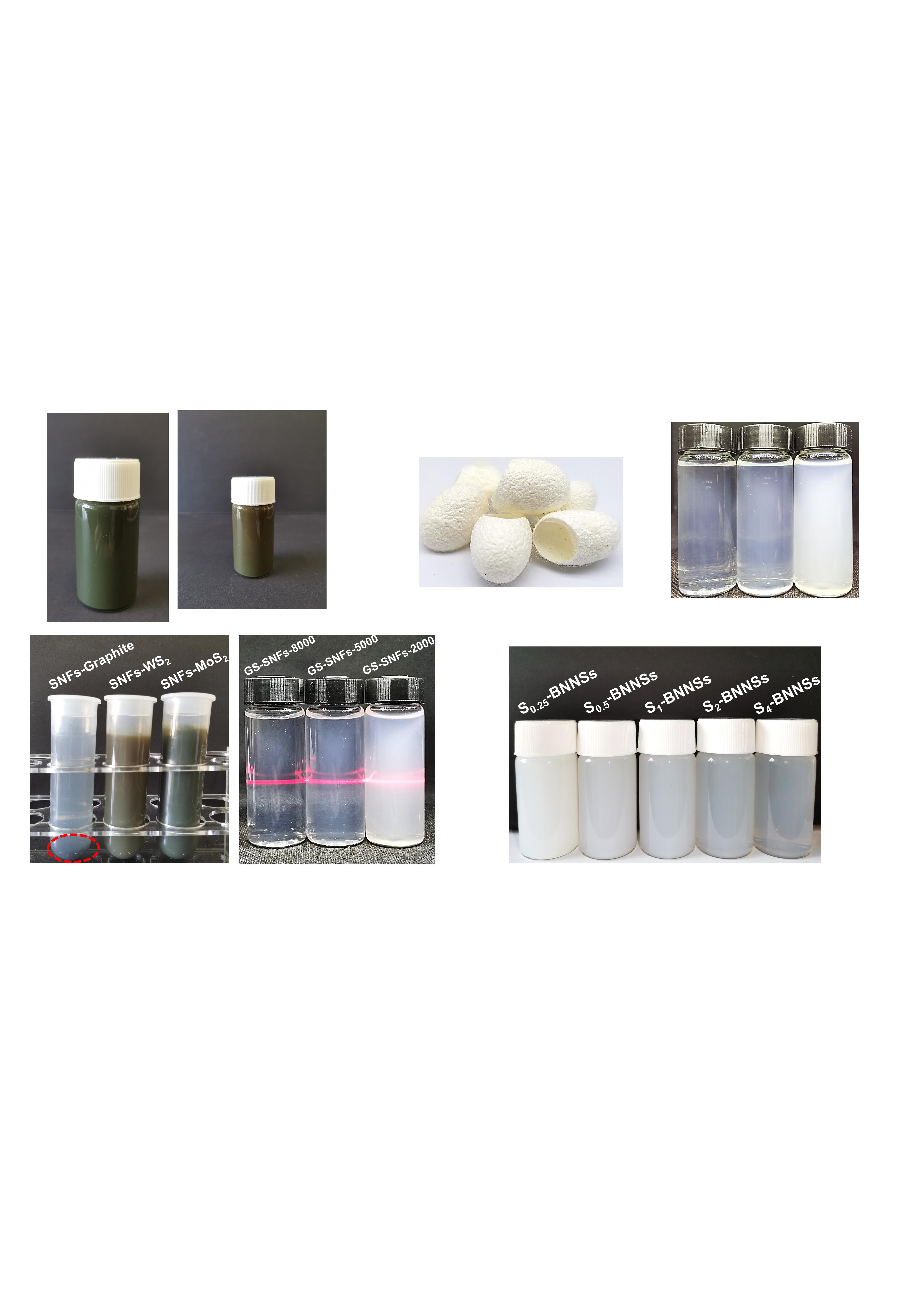


**Figure S7.** Photograph of S_n_-BNNSs dispersions prepared by SNFs-assisted exfoliation at different initial SNFs concentrations.

**Table S2.** The concentration of the S_n_-BNNSs dispersions, and the corresponding net BNNSs concentrations, BNNSs yield, and BNNSs mass ratio.

| Samples | Concentrations  (mg mL^−1^) | Net BNNSs concentration (mg mL^−1^) | BNNSs yield | BNNSs mass ratio (wt%) |
| --- | --- | --- | --- | --- |
| S_0.25_-BNNSs | 0.92 | 0.68 | 13.6% | 73.9 |
| S_0. 5_-BNNSs | 1.55 | 1.07 | 21.4% | 69.0 |
| S_1_-BNNSs | 2.21 | 1.31 | 26.2% | 59.3 |
| S_2_-BNNSs | 2.75 | 0.93 | 18.6% | 33.8 |
| S_4_-BNNSs | 4.48 | 0.75 | 15% | 16.7 |

**Figure S8.** TG curves of the h-BN, SNFs, and S_n_-BNNSs.

**Table S3.** Comparison on the yields of BNNSs using different liquid-phase exfoliation.

| Exfoliating agent | Solvent | Concentration of exfoliating agent (mg mL^-1^) | Ultrasonic power | Time (h) | Yield (%) | Reference |
| --- | --- | --- | --- | --- | --- | --- |
| Cellulose nanofibrils | Water | 0.5 | Not reported | 30 | 22% | ^[3]^ |
| Holocellulose nanofibrils | Water | 15 | 800 W | 2 | 23.4% | ^[4]^ |
| Thermo-responsive polymeric ionic liquids | Water | 2 | Not reported | 18 | 9.6% | ^[5]^ |
| Plant extract | Water | Not reported | 40 kHz | 24 | 23% | ^[6]^ |
| Silk nanofibrils | Water | 0.5 | 400 W | 8 | 21.4% | This work |
| Silk nanofibrils | Water | 1 | 400 W | 8 | 26.2% | This work |

**Table S4.** The yields and net concentrations of the corresponding nanosheets in the SNFs-assisted dispersions of different nanosheets, and the TG curves of bulk 2D-crystals, SNFs, S_1_-MoS_2_NSs, and S_1_-WS_2_NSs.

| Samples | Concentrations  (mg mL^−1^) | Net Nanosheet concentration  (mg mL^−1^) | Nanosheet yield | TG curve |
| --- | --- | --- | --- | --- |
| S_1_-MoS_2_NSs | 2.07 | 1.28 | 25.6% |  |
| S_1_-WS_2_NSs | 1.96 | 1.06 | 21.2% |  |


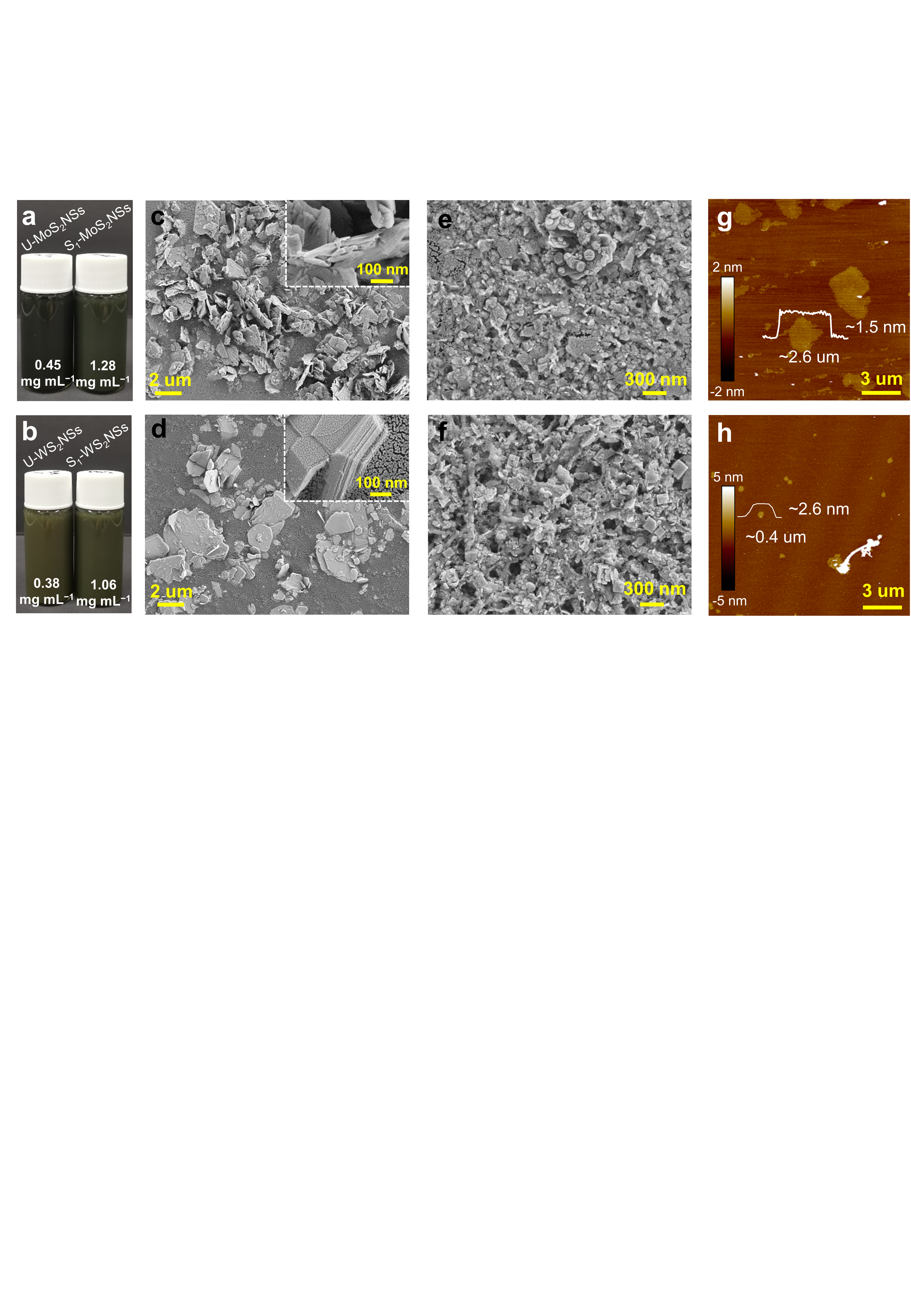


**Figure S9.** a, b) Photos of dispersions of MoS_2_ nanosheets and WS_2_ nanosheets prepared by SNFs-assisted exfoliation, U represented the exfoliated nanosheets without SNFs assistance. c, d) SEM images of bulk MoS_2_ and WS_2_. e, f) SEM images and g, h) AFM images of MoS_2_ nanosheets and WS_2_ nanosheets prepared by SNFs-assisted exfoliation.


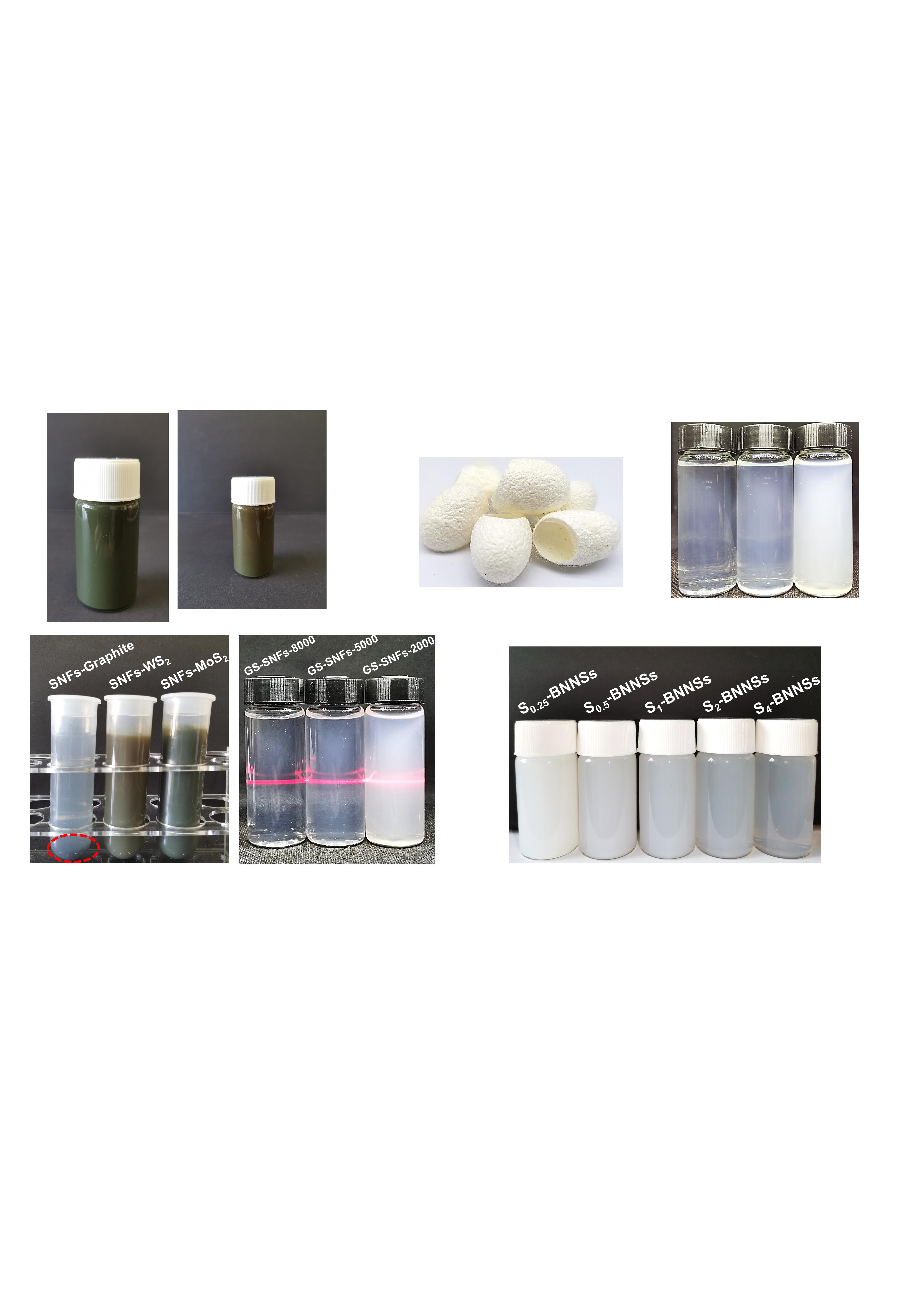


**Figure S10.** Photograph of SNFs-assisted exfoliated graphite, WS_2_, and MoS_2_ after centrifugation at 2000 rpm for 30 min (the un-fractured bulk crystal was marked by the red circle).





**Figure S11.** SEM image of SNFs/BNNSs-59.3% nanocomposite membrane.

**Figure S12.** The toughness of the SNFs/BNNSs membranes with different BNNS contents.

**Table S5.** Comparison on the in-plane *λ* of the SNFs/BNNSs membranes with those of the reported polymer-based BNNS nanocomposites.

| Fillers | Polymer matrix | Filler content (wt%) | In-plane *λ* (W/(m·K)) | Reference |
| --- | --- | --- | --- | --- |
| BNNS | Epoxy (EP) | 10 | 1.16 | ^[7]^ |
| Graphene/BNNS | Polyimide (PI) | 38.2 | 2.11 | ^[8]^ |
| BNNS | Polystyrene (PS) | 67.6 | 1.1 | ^[9]^ |
| Al_2_O_3_/BNNS | Silicone rubber (SR) | 30 | 2.78 | ^[10]^ |
| BNNS | Natural rubber (NR) | 30.8 | 2.08 | ^[11]^ |
| BNNS | Poly(vinylidene fluoride) (PVDF) | 20 | 1.43 | ^[12]^ |
| BNNS | Polydimethylsiloxane (PDMS) | 50 | 2.3 | ^[13]^ |
| BNNS | Holocellulose (HCNF) | 40 | 2.73 | ^[4]^ |
| Polydopamine/BNNS | Aramid nanofiber (ANF) | 50 | 3.3 | ^[14]^ |
| BNNS | SNFs | 33.8 | 2.92 | This work |
| BNNS | SNFs | 59.3 | 3.84 | This work |

**References**

[1] C. Guo, J. Zhang, X. Wang, A. T. Nguyen, X. Y. Liu, D. L. Kaplan, *Small* **2017**, 13, 1702266.

[2] Q. Niu, Q. Peng, L. Lu, S. Fan, H. Shao, H. Zhang, R. Wu, B. S. Hsiao, Y. Zhang, *Acs Nano* **2018**, 12, 11860.

[3] Y. Li, H. Zhu, F. Shen, J. Wan, S. Lacey, Z. Fang, H. Dai, L. Hu, *Nano Energy* **2015**, 13, 346.

[4] C. Zhang, M. Wang, X. Lin, S. Tao, X. Wang, Y. Chen, H. Liu, Y. Wang, H. Qi, *Carbohydrate Polymers* **2022**, 291, 119578.

[5] X. Wang, P. Wu, *ACS Applied Materials & Interfaces* **2018**, 10, 2504.

[6] A. R. Deshmukh, J. W. Jeong, S. J. Lee, G. U. Park, B. S. Kim, *ACS Sustainable Chemistry & Engineering* **2019**, 7, 17114.

[7] M. W. Akhtar, J. S. Kim, M. A. Memon, M. M. Baloch, *Composites Science and Technology* **2020**, 195.

[8] K. Kim, M. Kim, J. Kim, *Composites Science and Technology* **2014**, 103, 72.

[9] X.-B. Wang, Q. Weng, X. Wang, X. Li, J. Zhang, F. Liu, X.-F. Jiang, H. Guo, N. Xu, D. Golberg, Y. Bando, *Acs Nano* **2014**, 8, 9081.

[10] H. Yan, X. Dai, K. Ruan, S. Zhang, X. Shi, Y. Guo, H. Cai, J. Gu, *Advanced Composites and Hybrid Materials* **2021**, 4, 36.

[11] Z. Kuang, Y. Chen, Y. Lu, L. Liu, S. Hu, S. Wen, Y. Mao, L. Zhang, *Small* **2014**, 11, 1655.

[12] C. Du, M. Li, M. Cao, S. Song, S. Feng, X. Li, H. Guo, B. Li, *ACS Applied Materials & Interfaces* **2018**, 10, 34674.

[13] L. Ren, X. Zeng, R. Sun, J.-B. Xu, C.-P. Wong, *Chemical Engineering Journal* **2019**, 370, 166.

[14] T. Ma, Y. Zhao, K. Ruan, X. Liu, J. Zhang, Y. Guo, X. Yang, J. Kong, J. Gu, *ACS Applied Materials & Interfaces* **2019**, 12, 1677.
